# Angiotensin Dysregulation in Patients with Arterial Aneurysms

**DOI:** 10.3390/ijms26041502

**Published:** 2025-02-11

**Authors:** Maria Elisabeth Leinweber, Corinna Walter, Afshin Assadian, Chantal Kopecky, Oliver Domenig, Johannes Josef Kovarik, Amun Georg Hofmann

**Affiliations:** 1 Department of Vascular and Endovascular Surgery, Clinic Ottakring, 1160 Vienna, Austria; 2 Clinical Division of Nephrology and Dialysis, Department of Internal Medicine III, Medical University of Vienna, 1090 Vienna, Austria; 3 Attoquant Diagnostics, 1110 Vienna, Austria

**Keywords:** aneurysm, aorta, angiotensin, renin–angiotensin system

## Abstract

Besides playing a critical role in maintaining cardiovascular homeostasis, the renin–angiotensin–aldosterone system (RAS) has been strongly implicated in (aortic) aneurysm pathogenesis. This study aims to investigate systemic and local levels of angiotensin (Ang) and its metabolites in patients with arterial aneurysms, predominantly abdominal aortic aneurysms, using advanced biochemical profiling techniques to provide new insights into the involvement of RAS in aneurysm genesis. A prospective, single-center study was conducted between October 2023 and July 2024. Serum Ang metabolite levels were measured using RAS Fingerprint technology. Aortic tissue samples were analyzed for local RAS activity, including Ang levels and enzyme activity. Additionally, pre- and postoperative serum samples were obtained in a select group of patients. In total, 37 aneurysm patients and 56 controls were included. Aneurysm patients exhibited higher systemic levels of nearly all Ang metabolites compared to controls, with significant differences in Ang I (*p* = 0.002), Ang II (*p* = 0.047), Ang 1–5 (*p* = 0.004), and Renin (*p* = 0.014) in patients without pharmacological RAS interference. Aneurysm patients receiving ACE inhibitors showed lower serum concentrations in ACE2 activity (*p* = 0.042) and increased Ang IV levels (*p* = 0.049) compared to controls. Postoperative measurements indicated different dynamics regarding angiotensin metabolite changes in patients with or without ACE inhibition. This study provides the first comprehensive characterization of RAS profiles in aneurysm patients. These findings add to the body of evidence regarding associations between of RAS and the pathogenesis of arterial aneurysms.

## 1. Introduction

Despite major advances in the understanding of the pathophysiology of abdominal aortic aneurysms (AAA) and significant improvements in its surgical management, there remains considerable uncertainty regarding pharmacological approaches to minimize and prevent aneurysm progression and rupture [1,2,3]. Along with age, gender, smoking, ethnicity, and family history, hypertension is one of the most prominent risk factors for the development of AAA, with hypertensive patients having a relative risk of 1.66 for developing AAA compared to normotensive individuals [4,5,6,7]. The renin–angiotensin–aldosterone system (RAS) is a key physiological regulator of blood pressure. Besides playing a critical role in maintaining cardiovascular homeostasis, RAS has been strongly implicated in AAA pathogenesis, mainly through studies in murine models, where subcutaneous angiotensin (Ang) II infusion is a well-established method for inducing both abdominal and thoracic aortic aneurysms [8,9,10,11]. Interestingly, the development of aneurysms in these models appears to be independent of the hypertensive effects of Ang II. Other mediators that elevate blood pressure, such as norepinephrine, do not induce aneurysm formation, suggesting a unique role for Ang II beyond its hypertensive action [8].

Several mechanisms have been proposed to explain how Ang II contributes to aneurysm development and progression. Key mechanisms involve oxidative stress by stimulation of the production of reactive oxygen species, upregulation of the expression of matrix metalloproteinases, inflammation, and involvement in vascular smooth muscle cell apoptosis [12,13,14].

In the late 1980s, two alternative pathways of RAS were discovered. Ang 1–7, acting through the Mas receptor, as well as Ang II, acting through the Angiotensin II receptor type 2 (ATR2), antagonize the effects of the canonical angiotensin-converting enzyme (ACE)–Ang II–ATR1 axis, resulting in a beneficial effect for cardiovascular diseases by anti-inflammatory responses, vasodilation, anti-apoptosis, and anti-fibrosis [15,16,17,18].

Despite mounting evidence for the role of RAS in AAA progression, the intricate interactions among RAS peptides and enzymes in aneurysm formation remain inconclusively understood. The dynamic nature of RAS, characterized by the continuous formation and degradation of Ang metabolites, poses challenges for accurately quantifying these peptides in biological samples [19]. RAS Fingerprint is a novel diagnostic assay that enables comprehensive biochemical profiling of RAS components in plasma, serum, and tissue, offering insights into the activation state and enzymatic activity of Ang and 6 of its metabolites. It thereby circumnavigates limitations of single-molecule-targeted quantifications.

To date, no studies have specifically characterized RAS profiles in arterial aneurysm patients. Therefore, this study aims to investigate systemic and local aortic tissue levels of Ang and its metabolites in patients with arterial aneurysms, with and without pharmaceutical interference in the RAS via ACE inhibitors, using RAS Fingerprint to provide new insights into RAS involvement in arterial aneurysms.

## 2. Results

### 2.1. Sample Characteristics

Between October 2023 and July 2024, 59 patients were enrolled in this study, including 28 patients with abdominal aortic aneurysms (AAA), 6 patients with multiple central and/or peripheral aneurysms, 1 isolated common iliac artery aneurysm, and 2 patients with isolated peripheral aneurysms. In the control group, serum samples were collected from 6 varicose vein patients and 5 patients with carotid artery stenosis. Additionally, 45 chronic kidney disease controls were obtained as described in the Methods and Materials section. Overall, 19 patients (65.5%) in the AAA group and 49 patients (87.5%) in the control group were receiving ACE inhibitors (ACEi). Baseline sample characteristics are summarized in Table 1.

### 2.2. Systemic Angiotensin Quantification

**Figure 1:**
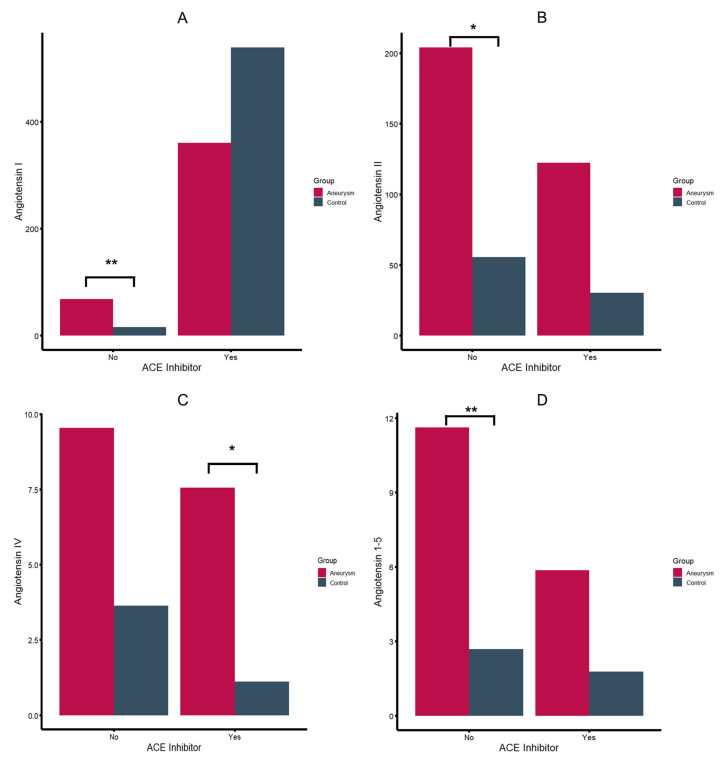
Angiotensin I (**A**), II (**B**), IV (**C**) and angiotensin 1–5 (**D**) serum levels in pmol/L (*: *p*-value < 0.05, **: *p*-value < 0.01).

Patients were stratified based on their registered use of ACEi since the pharmacological interference in the RAS limits comparative analyses between ACEi and ACEi naïve patients. As shown in Table 2, aneurysm patients exhibited a distinct RAS profile, compared to the control group. In patients without registered ACEi, aneurysm patients showed higher serum levels of all angiotensin (Ang) metabolites, with statistically significant differences in Ang 1–5 (*p*-value = 0.004), Ang I (*p*-value = 0.002), and angiotensin II (*p*-value: 0.047) (Figure 1). While not statistically significant, considerable differences were found regarding Ang IV (*p*-value = 0.095) and ACE (*p*-value = 0.064) Similar findings with higher Ang II, III, and IV concentrations were observed in patients with registered ACEi, even though statistically significant results were only obtained for Ang IV (Figure 1). Additionally, aneurysm patients under ACEi had lower ACE2 activity (*p*-value = 0.042) and slightly higher ACE activity (*p*-value = 0.067).

**Figure 2:**
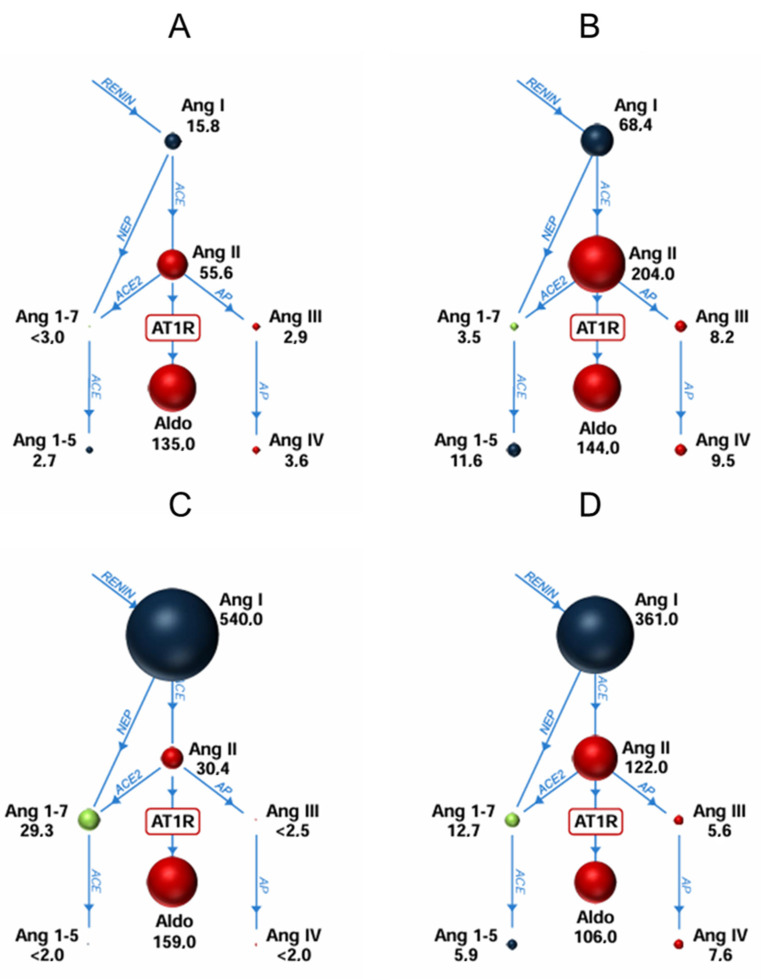
Schematic renin-angiotensin-aldosterone profiles for different cohorts ((**A**) controls without ACE inhibitors (ACEi), (**B**) aneurysm patients without ACEi, (**C**) controls with ACEi, (**D**) aneurysm patients with ACEi). The sizes of the spheres are adjusted to represent the respective serum levels.

Contrary to patients without ACE inhibition, aneurysm patients showed lower Ang 1–7 levels as controls, with a shift to an increased level of Ang 1–5 (5.9 vs. 1.8, *p*-value = 0.061). Despite the overall elevated Ang levels in aneurysm patients, no significant differences in aldosterone levels were observed compared to the control group, though there was a tendency towards lower aldosterone concentrations in aneurysm patients with ACEi. Regarding RAS enzyme activity, aneurysm patients receiving ACEi showed a significantly lower ACE2 activity compared to controls with ACEi (*p*-value = 0.042). Elevated levels of renin were observed for aneurysm patients compared to controls in the group of patients without ACEi (*p*-value = 0.014) (Table 2, Supplementary Figure S1) Schematic RAS profile illustrations for different groups are shown in Figure 2. Stratifying arterial aneurysm patients by type showed that RAS profiles of popliteal and iliac artery aneurysm patients (n = 3) resembled controls and that group differences are driven by AAAs and patients with multiple arterial aneurysms. Associations between aneurysm diameters in AAAs and RAS metabolites were not observed.

### 2.3. Changes in RAS Profiles After Intervention

**Figure 3:**
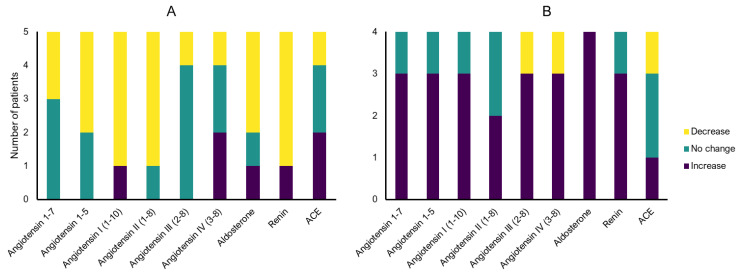
Angiotensin metabolite and enzyme serum levels pre- vs. post-surgery changes within the same patient ((**A**) patients with ACE inhibitors (ACEi), (**B**) patients without ACEi) (equal defined as ratio of 0.8–1.2).

In the matched analysis of pre- and postoperative samples, changes in postoperative RAS profiles were observed for all Ang metabolites, aldosterone, and renin. Postoperatively, most of the patients with ACEi showed declining levels of nearly all Ang metabolites as well as aldosterone and renin, whereas patients without ACE inhibitors mostly exhibit increasing levels of Ang metabolites, aldosterone, and renin (Figure 3). As part of a sensitivity analysis, pooled mean levels of all metabolites and enzymes were calculated (Table 3). Similar to the matched analysis, decreasing mean serum levels of Ang 1–7, Ang I, and aldosterone were observed in aneurysm patients treated with ACEi, and decreasing levels of Ang 1–5, Ang II, Ang III, and Ang IV were observed in aneurysm patients without ACEi after surgical or endovascular treatment. However, in contrast to patients with ACEi, elevated postoperative serum levels of aldosterone were observed in patients without ACEi.

### 2.4. Tissue Samples

Tissue samples from 4 patients with aortic aneurysms and 4 patients with carotid artery stenosis were obtained. RAS enzymes chymase, ACE, and ACE2 activities are shown in Supplementary Table S1. Enzyme activity could only be incompletely quantified in 2 carotid artery samples. In aortic tissues, enzyme activity and Ang II concentrations diverged between specimens. While the sample size is limited, it was observed that tissues of patients under ACEi (specimens 2 and 4) had higher levels of local chymase and ACE2 activity as well as higher concentrations of local Ang II compared to samples without systemic ACEi (specimens 1 and 3).

## 3. Discussion

Our finding that aneurysm patients showed higher serum levels of nearly all Ang metabolites aligns with previous findings, suggesting RAS overactivation might be associated with aneurysm development, independent of its hypertensive effects [20,21,22]. This is further evidenced by higher serum concentrations of alternative Ang metabolites and renin in aneurysm patients without recorded arterial hypertension (and therefore no pharmaceutical interference in the RAS). Patients with isolated popliteal or iliac artery aneurysms had RAS profiles that resembled controls. The fact that patients with AAAs or multiple arterial aneurysms had an upregulated RAS activity could hint at a dose–response relationship. While different mechanistic paths might also be involved, it should be noted that two patients with multiple aneurysms had no AAA but a mixture of iliac, femoral, or popliteal artery aneurysms.

We opted for a heterogenous control group consisting of patients with atherosclerotic disease, varicose veins, and chronic kidney disease on purpose to explore whether patients with aneurysmatic disease are characterized by a distinct RAS profile. That the RAS plays a significant role in a vast array of cardiovascular [23] and renal pathologies [24] is well researched by now. However, we aimed to investigate not only whether RAS profiles in aneurysm patients were different from healthy individuals but rather if they were specific to their condition and could therefore be distinctly identified from a pool of cardiovascular and kidney disease patients. The fact that we discovered significant differences in multiple RAS peptides between aneurysm patients and the mixed control group (both with and without pharmacological RAS interference) is a valuable indicator that aneurysm patients not only show RAS aberrations in general but also a specific (aneurysm-associated) RAS profile. This fits well with the vast amount of evidence regarding associations of selected RAS peptides and aneurysms.

The most investigated RAS metabolite in the context of arterial aneurysms is Ang II, which plays a pivotal role in various signaling pathways associated with cardiovascular function and dysfunction [25,26,27]. Ang II promotes growth in cardiac myocytes, fibroblasts, and vascular smooth muscle cells, contributing to cardiovascular remodeling through its interaction with the angiotensin I receptor (AT1R) [25,28]. Additionally, the identification of reactive oxygen species generated via the NADPH oxidase pathway as critical second messengers of AT1R further cemented Ang II’s role in cardiovascular remodeling and inflammation [29,30]. In our study, aneurysm patients exhibited mean Ang II levels that were four times higher than those in the control group. Interestingly, this finding persisted regardless of the use of ACEi in the patient groups. The interpretation of this result is complex due to various factors that may have influenced this observation. First, therapeutic adherence and the effective serum concentrations of ACEi were not monitored in this study. Additionally, ACE gene polymorphisms could have affected the efficacy of ACEi in some patients. Moreover, alternative ACE-independent pathways for local Ang II production may have contributed to the elevated Ang II levels. Ang substrates can be converted to Ang II, Ang III, and Ang IV in various tissues. For example, chymase-mediated Ang II pathways have been investigated as a relevant cause of ACEi escape in different cardiovascular diseases [31,32].

Corresponding to the higher serum levels of Ang II, downstream Ang III and Ang IV were also elevated in aneurysm patients. While differences in enzyme activity could in theory lead to diverging concentrations of downstream metabolites, the present results do not provide sufficient evidence for this hypothesis. Ang III, which can be synthesized from Ang II or Ang I, shows similar properties as Ang II, affecting also the AT1R and AT2R. It is believed that Ang III has a comparable effect on blood pressure and aldosterone release but may be different regarding inflammatory effects [26,33]. Ang IV is synthesized by aminopeptidase N from Ang III and binds on the AT4R, which can be found in various organs, including the kidneys, lungs, brain, and heart. The function of Ang IV is not conclusively understood. However, it has been reported that Ang IV and AT4R play a role in cognitive functions and anti-inflammatory processes, even if the effect on arterial blood pressure remains to be a matter of investigation [26,34,35]. Nevertheless, the influence of Ang III and Ang IV on vascular changes associated with aneurysm genesis is yet to be characterized. Of note, despite increased levels of Ang II, which binds at the AT1R, stimulating aldosterone release, no significant group differences in aldosterone serum concentrations were observed.

Additionally, we observed a significant elevation in Ang 1–5 levels in aneurysm patients but diverging results regarding Ang 1–7. Ang 1–7 is recognized for its counter-regulatory effects against Ang II, exerting vasodilatory, anti-inflammatory, and anti-fibrotic actions primarily through the Mas receptor [36]. Ang 1–7 has also been shown to suppress Ang II-induced aortic dilatation in experimental studies [17,37,38]. Ang 1–5, which is a more stable metabolite of Ang 1–7, has been less extensively studied but is believed to share some of the beneficial effects of Ang 1–7 [39,40]. However, whether the increased levels of these metabolites reflect an upregulation of the alternative RAS pathways in an attempt to restore vascular homeostasis or simply the extension of an increased RAS activity requires further investigation.

What has been less explored previously are changes in RAS after invasive aneurysm treatment. In a limited sample of 9 patients, patients without ACEi showed a tendency towards RAS upregulation postoperatively, whereas ACEi-naive patients had a stable or decreasing RAS activity. Pooling samples that were collected pre- and postoperatively resulted in no statistically significant group differences. It remains to be investigated whether treatment consistently affects systemic RAS profiles, but local Ang II production in diseased tissue, for instance, is at least discussed to have measurable systemic effects.

In our study, local RAS activity was shown within vascular tissues. Although Ang levels in most tissue samples were below detection limits, enzyme activity data for chymase, ACE, and ACE2 suggest active RAS components in both aneurysmal and stenotic tissues. This study represents the first instance of using the RAS Fingerprint assay in aneurysmatic aortic tissue. However, the sample size and enzyme activity below quantification thresholds in carotid artery tissue limit the interpretation thereof, particularly in the absence of a healthy control group, i.e., healthy aortic tissue.

This study represents an initial exploration of distinct RAS profiles in patients with arterial aneurysms. While excessive AT1R stimulation has been suggested to play a role in the development of AAA, translating these findings to clinical practice has proven challenging. Traditional approaches to limit AT1R activation (AT1R antagonists, ACEi) have not convincingly slowed AAA progression in clinical studies, and novel pharmacological approaches like direct renin inhibitors, chymase inhibitors, and AT2R agonists have not yet been transferred from preclinical to clinical studies [2,41,42,43]. SGLT-2 inhibitors have been shown to ameliorate aneurysm progression in animal models and alter RAS profiles at least in specific patient cohorts [44]. It remains to be investigated whether these results can be consistently replicated in patients, but this could be a further indicator for the involvement of RAS in arterial aneurysms and a potential pharmaceutical target.

### Limitations

The study’s observational and single-center design, along with patient selection, may have introduced selection bias, potentially limiting the external validity of the results. Additionally, the small sample size, particularly within certain subgroups such as patients who underwent surgical intervention or had isolated peripheral aneurysms, poses a relevant limitation. Moreover, therapy compliance with ACEi was not controlled, which could have influenced Ang levels in patients, complicating the interpretation of the data. Another notable limitation is the limited quantifiability of Ang metabolites and enzyme activity in tissue samples. Furthermore, the absence of a control group for tissue samples restricts the ability to compare aneurysmal tissue findings with those from normal aortic tissue. In summary, the produced results should be considered preliminary based on the exploratory design of the present study and require validation in larger cohorts.

## 4. Materials and Methods

### 4.1. IRB Approval

The study was approved by the appropriate institutional review board and ethics committee of the City Government of Vienna (ID: EK 21-135-VK). Before study inclusion, patients had to give written and informed consent. The study was conducted according to the Declaration of Helsinki.

### 4.2. Design and Study Participants

This was a prospective, single-center study performed at the Department of Vascular and Endovascular Surgery, Klinik Ottakring Vienna, between 10/2023 and 07/2024. Patients affected by arterial aneurysms, predominantly abdominal aortic aneurysms, were included. Patients with a prescribed angiotensin II type 1 receptor (AT1R) antagonist or SGLT-2 inhibitor were excluded from participation. Serum was collected from all patients, while aortic tissue was collected from patients undergoing open surgical repair of AAA, and atherosclerotic carotid samples were collected from patients undergoing carotid endarterectomy.

### 4.3. Material Harvested and Tissue Preparation

Venous blood samples were taken at enrolment and subsequently centrifuged at 4000 rpm for 15 min to obtain serum. Serum was aliquoted into two 0.5 mL and two 1 mL collection tubes and stored at −20 °C until analysis. For 9 aneurysm patients, pre- and postoperative serum samples were available. In none of these patients were changes in the prescription of ACE inhibitors between the two time points of serum sampling documented.

Aortic tissue was obtained during surgery by excision of a small flap of tissue from the aneurysmal part of the aorta. Carotid artery tissue was harvested and collected from patients who underwent carotid endarterectomy. Calcified portions of the carotid tissue were removed as much as feasible to retain only viable vascular tissue. The tissue was thoroughly rinsed with saline to remove blood and transported at 4 °C from the operating theater to the local laboratory, where it was snap frozen and stored at −80 °C.

### 4.4. RAS Equilibrium Analysis and Enzyme Activities

Serum samples were analyzed in a commercial diagnostic laboratory for RAS Fingerprint analysis using previously validated and described methods [45,46,47,48]. This includes 6 angiotensin metabolites and aldosterone, as well as surrogates for enzyme activities. Angiotensin-converting enzyme 2 and neprilysin (NEP) were separately quantified. Evaluation of RAS enzyme activities in tissues (as performed in aortic tissue and carotid artery samples) has previously been described [49] and follows the same approach as in serum. Please refer to the Supplementary Methods for details.

### 4.5. Controls

Serum samples were collected from 6 varicose vein patients as well as 5 patients with carotid artery stenosis. Additionally, 45 controls from a recent randomized controlled prospective study investigating the effect of 12 weeks of treatment with the SGLT-2 inhibitor empagliflozin, on top of ACE inhibitors, on the molecular RAS dynamics in 23 diabetic and 22 non-diabetic patients with chronic kidney disease were obtained from the corresponding author and included in the analysis [50]. We exclusively used the specimen collected prior to SGLT-2 inhibitor therapy for the present analysis. None of the varicose vein controls were on ACE inhibitors, whereas all other control patients except for one (those with carotid stenosis and chronic kidney disease) received ACE inhibitors. By utilizing a mixed control group, we were able to assess differences between arterial aneurysm patients and other arterial and venous pathologies as well as patients with a cardiovascular risk profile. We aimed to investigate whether aneurysm patients would be characterized by a distinct RAS profile compared to a heterogeneous group of patient controls, i.e., whether aneurysm patients could be detected by their RAS profile out of a pool of cardiovascular patients.

### 4.6. Analysis

Descriptive statistics included an investigation of central tendency and dispersion metrics, including mean and median as well as standard deviation and interquartile range. Group means were either compared via *t*-test or Wilcoxon–Mann–Whitney test. Analyses were conducted in the full cohort as well as different strata, including aneurysms and controls or pre- and post-operative samples. Multiple testing correction was not performed. The underlying statistical assumption of multiple testing correction is multiple hypotheses to be tested. In a physiological model such as the RAS, where metabolites are interconnected in a flow model, this assumption would be violated. Therefore, the main hypothesis to be tested in the present case is that differences in RAS activity and *p*-values of the respective metabolites should be evaluated based on this hypothesis. In 9 aneurysm patients, serum samples were obtained exclusively secondary to invasive treatment, in 9 pre- and post-operatively, and in the remaining 11 exclusively prior to surgery. For the primary analyses (aneurysms vs. controls), only the pre-operative samples were used for patients with two measurements. Regarding pre- and post-operative changes in systemic RAS profiles, two analyses were conducted. First, a matched analysis investigating changes within the same patient, and second, a pooled analysis of pre- and post-operatively obtained samples was performed. A *p*-value of <0.05 was considered as the threshold for statistically significant results. All statistical analyses were conducted using R version 4.10 (R Foundation for Statistical Computing, Vienna, Austria).

## 5. Conclusions

While a multitude of challenges persist in the conclusive investigation of the RAS and arterial aneurysms, RAS upregulation in general and elevated concentrations of alternative Ang metabolites are at the very least strongly associated with arterial aneurysms, if not causally linked. Even if pharmacological targets in the RAS have not been effective on a broader scale so far, these findings could prove clinically relevant in the future, as RAS profiles could serve as early predictors for patients at elevated risk for aneurysm development, potentially years before radiographic studies capture manifested pathologies. Nevertheless, developing a comprehensive picture of the interplay between the RAS and arterial aneurysms remains a challenge that warrants further investigation.

## Figures and Tables

**Table 1:** Baseline sample characteristics. Age is depicted as median (Q1–Q3). BMI is missing for 6 aneurysm patients (BMI: body mass index, PAD: peripheral arterial occlusive disease, CHD: coronary heart disease).

	ACE Inhibitor		No ACE Inhibitor	
	Aneurysm	Controls	Aneurysm	Controls
N	19	49	18	7
Age	71 (62–75)	67 (57–75)	76 (66–81)	52 (50–63)
Sex (m:f)	15 (78.9%):4 (21.1%)	32 (65.3%):17 (34.7%)	17 (94.4%):1 (5.6%)	2 (28.6%):5 (71.4%)
BMI	28.3 (4.8)	28.9 (3.8)	25.3 (3.7)	27.5 (4.0)
Arterial hypertension	19 (100%)	49 (100%)	9 (50.0%)	4 (57.1%)
Diabetes	2 (10.5%)	25 (51.0%)	6 (33.3%)	2 (28.6%)
PAD	4 (21.1%)	5 (10.2%)	4 (22.2%)	4 (57.1%)
CHD	9 (47.4%)	10 (20.4%)	2 (11.1%)	1 (14.3%)
Chronic kidney disease	8 (42.1%)	46 (93.9%)	4 (22.2%)	1 (14.3%)

**Table 2:** Results from the serum RAS Fingerprint and *p*-values from group comparisons using a *t*-test (ACE inhibitor group) or Wilcoxon–Mann–Whitney test (no ACE inhibitor). Angiotensins and aldosterone are given as pmol/L, neprilysin and ACE2 activity as (nmol/L)/h (ACEi: ACE inhibitor, *: statistically significant result).

	ACEi			No ACEi		
	Aneurysm	Controls	*p*-Value	Aneurysm	Controls	*p*-Value
N	19	49	-	18	7	-
Angiotensin 1–7	12.7	29.3	0.061	3.5	1.0	0.67
Angiotensin 1–5	5.9	1.8	0.061	11.6	2.7	0.004 *
Angiotensin I (1–10)	361	540	0.322	68.4	15.8	0.002 *
Angiotensin II (1–8)	122	30.4	0.122	204	55.6	0.047 *
Angiotensin III (2–8)	5.6	1.2	0.091	8.2	2.9	0.386
Angiotensin IV (3–8)	7.6	1.1	0.05	9.5	3.6	0.095
Aldosterone	106	159	0.147	144	135	0.745
Neprilysin	0.4	0.4	0.995	0.9	0.3	0.204
ACE2	1.1	1.5	0.042 *	1.5	1.1	0.458
ACE	0.9	0.3	0.067	2.8	4.5	0.064
Renin	483	570	0.64	273	71.5	0.014 *

**Table 3:** Pooled mean serum levels of angiotensin metabolites and enzymes in samples obtained before or after surgery/intervention, stratified by registered ACE inhibitors. No statistically significant differences were observed. Angiotensins and aldosterone are given as pmol/L.

	ACEi		No ACEi	
	Pre	Post	Pre	Post
N	15	9	13	9
Angiotensin 1–7	12.8	6.7	3.7	4.0
Angiotensin 1–5	4.9	5.5	13.8	8.9
Angiotensin I (1–10)	408	112	75.6	116
Angiotensin II (1–8)	86.0	126	251	190
Angiotensin III (2–8)	3.5	7.1	10.8	8.3
Angiotensin IV (3–8)	5.6	8.0	11.7	9.8
Aldosterone	121	106	152	287
Renin	494	241	326	206
ACE	0.6	1.5	2.8	2.6

## Data Availability

The original contributions presented in this study are included in the article/Supplementary Materials. Further inquiries can be directed to the corresponding author(s).

## Supplementary Materials

The supporting information can be downloaded at: https://www.mdpi.com/article/10.3390/ijms26041502/s1.
