# Supplementary material for: Angiotensin Dysregulation in Patients with Arterial Aneurysms
Source: Int J Mol Sci. 2025 Feb 11;26(4):1502. doi: 10.3390/ijms26041502 (PMC11855860; doi:10.3390/ijms26041502)
Supplement: Supplementary file 1 [file ijms-26-01502-s001.zip › ijms-3447790-supplementary.pdf]

Supplementary Figure S I. Serum concentrations in pmol/L of different RAS enzymes and metabolites, stratified by group and ACE inhibitor therapy). Angiotensins and aldosterone are given as pmol/L, neprilysin and ACE2 activity as (nmol/L)/h. (A: Renin, B: ACE 2, C: Angiotensin III, D: Angiotensin 1-7, E: Aldosterone, F: Neprilysin (NEP))

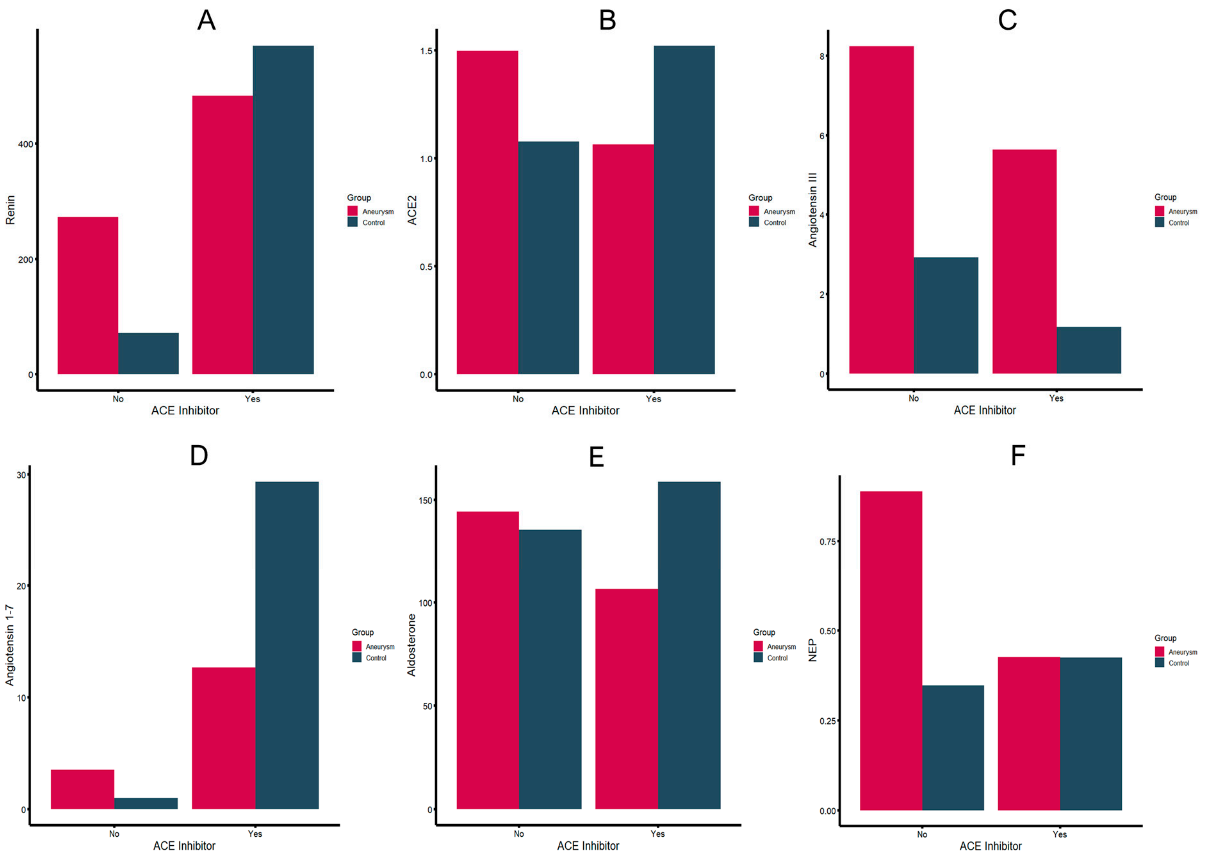

Supplementary Table SI. RAS enzyme activity of chymase, ACE, and ACE2 as well as angiotensin II (Ang II) in aortic and carotid artery tissue samples. Apart from Ang II no angiotensin levels could be measured in the respective samples.

	Chymase Activity Ang II Formation [pg/µg protein/h]	ACE Activity Ang II Formation [pg/µg protein/h]	ACE2 Activity Ang 1-7 Formation [pg/µg protein/h]	Ang II (1-8) [fmol/g]
**Aorta 1**	216,8	409,7	<5	<20
**Aorta 2**	1176,9	192,7	11,4	26,6
**Aorta 3**	262,0	447,3	9,7	<20
**Aorta 4**	1211,5	375,3	26,7	32,5
**Carotid 1**	201,3	450,9	<5	<20
**Carotid 2**	<100	<100	<5	<20
**Carotid 3**	<100	<100	<5	<20
**Carotid 4**	<100	144,1	<5	<20

Supplementary Methods

RAS Equilibrium Analysis

Serum samples were analyzed in a commercial diagnostic laboratory for RAS-Fingerprint analysis using previously validated and described methods.[1-4] RAS hormones quantified in the assay include: angiotensin I (Ang I), angiotensin II (Ang II), angiotensin III (Ang III), angiotensin IV (Ang IV), angiotensin 1-7 (Ang1-7), angiotensin 1-5 (Ang1-5), and aldosterone. Briefly, the assay was performed using equilibrium dialysis from serum samples that did not contain a protease inhibitor. The equilibrated serum samples were stabilized (ex vivo incubation at 37ºC for one hour) and spiked with stable isotope labeled internal standards for each angiotensin metabolite as well as with the deuterated internal standard for aldosterone (aldosterone D4) at a concentration of 200pg/mL. The samples then underwent C-18-based solid-phase-extraction and were subjected to LC-MS/MS analysis using a reversed-phase analytical column (Acquity UPLC C18, Waters) operating in line with a Xevo TQ-S triple quadrupolele mass spectrometer (Waters Xevo TQ/S, Milford, MA) in multiple reaction monitoring mode. Internal standards were used to correct for analyte recovery across the sample preparation procedure in each individual sample. Analyte concentrations were reported in pM and are calculated considering the corresponding response factors determined in appropriate calibration curves in sample matrix, when integrated signals exceeded a signal-to-noise ratio of 10. The lower limit of quantification was 3.0 pM for AngI, 2.0 pM for AngII, 3.0 pM for Ang1-7, 2.0 pM for Ang1-5, 2.5 pM for AngIII, 2.0 pM for AngIV, and 13,9 pM for aldosterone.

The following surrogate markers were calculated based on results of the RAS-Fingerprint analysis: plasma renin activity (PRA-S), ACE activity (ACE-S) and adrenal responsiveness (AA2-ratio). PRA-S, an index of plasma renin activity, was calculated as the sum of Ang I + Ang II. ACE-S, a measure of ACE enzyme activity, was calculated by dividing Ang II / Ang I. AA2, a measure of adrenal responsiveness to Ang II, was calculated as ALD / Ang II. All RAS analytes and PRA-S are in pmol/L. AA2 and ACE-S are ratios of pmol/L:pmol/L.

RAS Enzyme Activities (ACE2 / NEP) in serum

NEP activity was determined in diluted serum samples (phosphate-buffered saline, pH 7.4) after spiking samples with Ang I and ex vivo incubation at 37 °C in the presence (inhibitor) and absence (solvent) of the NEP inhibitor LBQ-657 (10 µmol/L, Sigma-Aldrich). Lisinopril (10 µmol/L, Sigma Aldrich), Aminopeptidase inhibitor (10 µmol/L, Sigma-Aldrich), Z-Pro-prolinal (10 µmol/L, Sigma-Aldrich) and ACE2 inhibitor MLN-4760 (10 µmol/L, Sigma-Aldrich) were added to all samples for substrate (Ang I) and product (Ang 1-7) stabilization. Quantification of

Ang I and Ang 1-7 was performed as described above by LC-MS/MS. Specific activity of NEP was calculated by determining the inhibitor sensitive fraction (solvent minus inhibitor) of Ang 1-7 formation.

ACE2 activity was measured in diluted serum (phosphate-buffered saline, pH 7.4) after spiking samples with Ang II and ex vivo incubation at 37 °C in the presence (inhibitor) and absence (solvent) of the ACE2 inhibitor MLN-4760 (10 µmol/L, Sigma-Aldrich). Lisinopril (10 µmol/L, Sigma Aldrich), Aminopeptidase inhibitor (10 µmol/L, Sigma-Aldrich) and Z-Pro-prolinal (10 µmol/L, Sigma-Aldrich) were added to all samples for substrate (Ang II) and product (Ang 1-7) stabilization. Quantification of Ang II and Ang 1-7 was performed as described above by LC-MS/MS. Specific activity of ACE2 was calculated by determining the inhibitor sensitive fraction (solvent minus inhibitor) of Ang 1-7 formation.

RAS Enzyme Activity (ACE / ACE2 / Chymase) in tissue

Evaluation of RAS enzyme activities in tissues (Aorta / Carotid) has previously been described[5] and follows the same approach as described above in "Enzyme Activities in serum".

References

1: Domenig O, Manzel A, Grobe N, Königshausen E, Kaltenecker CC, Kovarik JJ, Stegbauer J, Gurley SB, van Oyen D, Antlanger M, Bader M, Motta-Santos D, Santos RA, Elased KM, Säemann MD, Linker RA, Poglitsch M. Neprilysin is a Mediator of Alternative Renin-Angiotensin-System Activation in the Murine and Human Kidney. Sci Rep. 2016 Sep 21;6:33678. doi: 10.1038/srep33678. PMID: 27649628; PMCID: PMC5030486.

2: Larouche-Lebel É, Loughran KA, Huh T, Oyama MA. Effect of angiotensin receptor blockers and angiotensin converting enzyme 2 on plasma equilibrium angiotensin peptide concentrations in dogs with heart disease. J Vet Intern Med. 2021 Jan;35(1):22-32. doi: 10.1111/jvim.16025. Epub 2020 Dec 24. PMID: 33368659; PMCID: PMC7848346.

3: Guo Z, Poglitsch M, McWhinney BC, Ungerer JPJ, Ahmed AH, Gordon RD, Wolley M, Stowasser M. Measurement of Equilibrium Angiotensin II in the Diagnosis of Primary Aldosteronism. Clin Chem. 2020 Mar 1;66(3):483-492. doi: 10.1093/clinchem/hvaa001. PMID: 32068832.

4: Adin D, Atkins C, Domenig O, DeFrancesco T, Keene B, Tou S, Stern JA, Meurs KM. Renin-angiotensin aldosterone profile before and after angiotensin-converting enzyme-inhibitor administration in dogs with angiotensin-converting enzyme gene polymorphism. J Vet Intern Med. 2020 Mar;34(2):600-606. doi: 10.1111/jvim.15746. Epub 2020 Feb 29. PMID: 32112596; PMCID: PMC7097578.

5: Kaltenecker CC, Domenig O, Kopecky C, Antlanger M, Poglitsch M, Berlakovich G, Kain R, Stegbauer J, Rahman M, Hellinger R, Gruber C, Grobe N, Fajkovic H, Eskandary F, Böhmig GA, Säemann MD, Kovarik JJ. Critical Role of Neprilysin in Kidney Angiotensin Metabolism. Circ Res. 2020 Aug 14;127(5):593-606. doi: 10.1161/CIRCRESAHA.119.316151. Epub 2020 May 18. PMID: 32418507.
